# Editorial: Molecular and Cellular Underpinnings of Age-Related Memory Loss

**DOI:** 10.3389/fcell.2021.743187

**Published:** 2021-08-20

**Authors:** Stylianos Kosmidis, Christine A. Denny, Alex Dranovsky, Efthimios M. C. Skoulakis

**Affiliations:** ^1^Zuckerman Institute, Howard Hughes Medical Institute, Columbia University, New York, NY, United States; ^2^Department of Psychiatry, Columbia University Irving Medical Center (CUIMC), New York, NY, United States; ^3^Division of Systems Neuroscience, Research Foundation for Mental Hygiene, Inc. (RFMH)/New York State Psychiatric Institute (NYSPI), New York, NY, United States; ^4^Alexander Fleming Biomedical Sciences Research Center, Vari, Greece

**Keywords:** aging, cognitive decline, model organism, neurogenesis, therapeutic intervention

Our memories define who we are. Normal aging is often accompanied by a decline in memory functions leading to a condition known as age-related memory loss (ARML). With the aged population predicted to double in the next 30 years according to UN population dynamics (https://www.un.org/en/global-issues/ageing), ARML is expected to be a significant attenuator of life quality and elevate the financial burden of elderly care for families and society at large. Understanding the mechanisms underlying age-related memory decline is imperative for developing pharmaceutical interventions, which will ameliorate loss or restore cognitive functions in older individuals. Improving memory in the affected population will pave the road for restoring quality of life and alleviating dire socioeconomic consequences.

The collection of research articles herein provides novel contributions in the field of aging research, with specific focus on the molecular constituents of memory loss.

In the first article of this topic, Hahn et al. propose that the DNA methyltransferase DNMT1 function is implicated in age-related loss of cortical inhibitory interneurons. Interestingly, DNMT1-deficient mice exhibited improved sensory-motor performance and reduced aging-associated transcriptional changes, leading the authors to posit that the DNMT1 protein may act indirectly on interneuron survival in aged mice, potentially by modulating the proteostasis network.

To provide a link between aging and glucose metabolism, Ripoli et al. used a mouse model of type 1 diabetes and discovered that memory impairment related to aging appears associated with inhibition of the transcription factor cAMP-response element-binding protein (CREB). The authors show that experimentally induced hyperglycemia, can downregulate CREB phosphorylation and CREB-mediated mRNA expression of synaptic proteins in hippocampal primary neurons. Their findings highlighted interesting mechanisms underlying hyperglycemia-related memory loss and the necessity of further studying the role of glucose-driven CREB transcriptional activity in the process, as well as its potential impact on personalized medicine approaches.

Scott et al. studied the impact of the gut microbiome on hippocampal neurogenesis, contextual fear memory, and aging, providing a novel functional connection. Their results show that disruption of the gut microbiome can affect hippocampal neurogenesis in an age- and sex-dependent manner, suggesting that these changes can in principle alter the dentate gyrus functional network and, subsequently, memory-related processes in an age-dependent manner.

Moreover, Arredondo et al. summarize current knowledge on the roles of Wnt signaling and review data suggesting distinct roles for the canonical and non-canonical Wnt signaling cascades in the regulation of different stages of neurogenesis. Wnt signaling in fact, may be a highly conserved molecular pathway underlying aging in many species.

In accord, Inestrosa et al. used the Andean rodent *Octodon degus* (*O. degus*, common degu), often kept as a pet, to explore the age-related changes in the expression of key Wnt components. Their findings are in congruence with those from other species and suggest that the brain of *O. degus* can be used as model to study brain aging and its consequences.

In an effort to further delineate the molecular signatures of aging, Dunn et al. used a genetically diverse mouse population to characterize individual differences in cognitive abilities in adulthood, and to search for evidence of cognitive reserve and/or resilience in middle-aged mice. Using RNA-Sequencing, they present evidence nominating the Rho guanine nucleotide exchange factor-encoding gene Trio as a modulator of working memory ability, implicating the actin cytoskeleton in the process. More importantly, they propose that the usage of B6-BXD recombinant inbred lines, are a promising tool to study the molecular mechanisms of ARML before onset and translate these findings to humans.

This special issue also focused on diagnostic and therapeutic interventions for ARML. To this end, Cocco et al. propose that plasma levels of brain-derived neurotrophic factor (BDNF) can serve as a simple and low-cost diagnostic tool with several clinical applications. Using transcranial direct current stimulation (tDCS) in 3 × Tg-Alzheimer's disease (AD) mice, the authors showed that tDCS induced a significant increase of plasma BDNF levels in wild type mice, but not in 3 × Tg-AD mice. They also discuss the potential of identifying memory-related disorders in a pre-clinical stage, allowing more effective disease-modifying interventions.

In a similar fashion, Sun et al. propose that pro-BDNF protein is implicated in memory. Using a variety of molecular and behavioral assays the authors showed that blocking hippocampal pro-BDNF early in development plays a role in spatial cognition in adults. These findings are consistent with the hypothesis that postnatal pro-BDNF plays an essential role in synaptic and cognitive functions and can be used for therapeutic interventions to alleviate memory impairments and consequently ARML.

Lastly, Mhillaj et al. explored the possibility that celecoxib (CXB), a selective inhibitor of the pro-inflammatory cyclooxygenase-2 can have neuroprotective properties in subjects with early AD or mild cognitive impairment (MCI). Importantly, using *in vitro* methods, they showed that celecoxib modulates the heme oxygenase/biliverdin reductase (HO/BVR) system, counteracting the β-amyloid peptide (Aβ)-induced reactive oxygen species (ROS) production, lipid peroxidation, and the growth rate of Aβ oligomers with a mechanism dependent on Heme oxygenase −1.

In conclusion, we are confident that this Special Research Topic Issue contributes a significant amount of information regarding the molecular underpinnings of Age-Related Memory Loss. It is timely and of paramount importance to continue pushing the boundaries of aging research to promote ameliorative strategies that combat cognitive decline and improve the quality of life of the elderly.

## Author Contributions

SK, CD, AD, and ES wrote the manuscript. All authors contributed to the article and approved the submitted version.

## Conflict of Interest

The authors declare that the research was conducted in the absence of any commercial or financial relationships that could be construed as a potential conflict of interest.

## Publisher's Note

All claims expressed in this article are solely those of the authors and do not necessarily represent those of their affiliated organizations, or those of the publisher, the editors and the reviewers. Any product that may be evaluated in this article, or claim that may be made by its manufacturer, is not guaranteed or endorsed by the publisher.

